# Could Circulating Tumor Cells and ARV7 Detection Improve Clinical Decisions in Metastatic Castration-Resistant Prostate Cancer? The Istituto Nazionale dei Tumori (INT) Experience

**DOI:** 10.3390/cancers11070980

**Published:** 2019-07-13

**Authors:** Pierangela Sepe, Elena Verzoni, Patrizia Miodini, Melanie Claps, Raffaele Ratta, Antonia Martinetti, Roberta Mennitto, Elisa Sottotetti, Giuseppe Procopio, Vera Cappelletti, Maria Grazia Daidone

**Affiliations:** 1 Department of Medical Oncology, Fondazione IRCCS Istituto Nazionale dei Tumori di Milano, 20133 Milano, Italy; 2 Biomarker Unit, Department of Applied Research and Technological Development Fondazione IRCCS Istituto Nazionale dei Tumori di Milano, 20133 Milano, Italy

**Keywords:** castration-resistant prostate cancer, abiraterone, enzalutamide, androgen receptor splicing variants, circulating tumor cells, liquid biopsy, precision medicine

## Abstract

Enzalutamide and abiraterone have been shown to improve progression-free survival (PFS) and overall survival (OS) in metastatic castration-resistant prostate cancer (mCRPC) patients. Moreover, some patients may not benefit from the inhibition of androgen receptor (AR) activity or, alternatively, may develop secondary resistance. Detection in patients’ circulating tumor cells (CTCs) of ARV7, a splicing variant of AR lacking the ligand-binding domain, showed a link with treatment failure. Independent confirmation of the predictive role of CTC status combined with ARV7 detection is, therefore, a priority for extending personalized biomarker-driven treatments to all patients. In this prospective observational study, CTC status and the expression of AR and ARV7 were measured in 37 mCRPC patients, before starting treatment with enzalutamide or abiraterone, by employing commercially available kits. CTC status was positive in 21/37 patients: 46% and 24% of CTC-positive patients were defined as AR- and ARV7-positive, respectively. Kaplan–Meier estimates showed that positivity for each variable was significantly associated with poorer radiological PFS, PSA-PFS, and OS. All considered treatment outcomes worsened when going from CTC-negative to CTC-positive/ARV7-negative to CTC-positive/ARV7-positive patients, both in the global case series and in patients stratified into three groups based on basal PSA levels. Presently, technical approaches appear to be mature for introducing CTC/ARV7 tests in clinical practice.

## 1. Introduction

Metastatic castration-resistant prostate cancer (mCRPC) is a hormone-driven disease, the progression of which is promoted by the androgen receptor (AR) signal despite low or undetectable serum androgen levels. New generation hormonal agents (NHA), such as enzalutamide and abiraterone acetate, have been shown to improve survival in mCRPC patients by suppressing androgenic synthesis or targeting the androgen receptor directly [1,2,3,4].

Despite recent progress and the approval of new drugs, mCRPC is still associated with a high rate of progression and death. The absence of biomarkers predictive of clinical outcome and treatment response makes it challenging to treat this disease and choose the best treatment sequence.

Moreover, a proportion of patients (20–40%) treated with new hormonal agents may not benefit from them or, alternatively, may develop secondary resistance after the initial disease response [5].

Understanding the mechanisms underlying primary or acquired resistance represents an unmet need, as well as a challenge for clinicians for finding alternative therapies or the best treatment sequence with the aim of improving patients’ clinical outcomes. Multiple mechanisms regarding androgen receptor altered signaling could be involved in the resistance to hormonal therapies and the poor prognosis of mCRPC. They include, for instance, an increased steroidogenesis secondary to the overexpression of CYP17A1; activation of alternative growth factor; cytokine signaling cascades, such as phosphoinositide 3-kinase/AKT serine/threonine kinase (AKT), mitogen-activated protein kinase (MAPK), Janus kinase (JAK)/signal transducer, and activator of transcription (STAT); and loss or increase of androgen receptor expression, androgen receptor gene mutations or a constitutive activation of receptor splice variants [6,7,8,9,10].

Several androgen receptor variants have been identified, but the most commonly studied is the androgen receptor splice variant 7 (ARV7) [11]. This splice variant encodes a truncated androgen receptor protein that lacks the ligand-binding domain (target of enzalutamide and abiraterone), resulting in a constitutive activation in the absence of its ligand [9].

Directly involved in hematologic dissemination, circulating tumor cells (CTCs) permit tumor-derived material to be obtained without invasive tissue biopsies. In particular, CTC enumeration and change in CTC counts during treatment are prognostic and predictive biomarkers of response in mCRPC patients [12]. Lower CTC counts at baseline are associated with an improved progression-free survival (PFS) and overall survival (OS) in mCRPC patients treated with abiraterone [13,14,15]. AR and its splicing variants are the most studied molecular features in CTCs isolated in patients with mCRPC, and many works have investigated their clinical relevance [16]. Other less studied features of unclear clinical validity are gene fusions, such as TMPRSS2-ERG and loss of PTEN [17,18].

Five years ago, Antonarakis et al. hypothesized that the detection of ARV7 messenger RNA in CTCs from men with mCRPC receiving NHA may predict response or resistance to agents directed against the androgen receptor [19,20]. The detection of ARV7 is, in fact, associated with shorter radiographic PFS (rPFS) and poor PSA responses; however, without strong evidence of a causal role in mediating this resistance, it remains possible that ARV7 is a marker of more advanced disease or a higher disease burden. Several studies involving patients with mCRPC have shown that androgen receptor variants are often expressed in metastatic tissue and associated with faster disease progression and poor prognosis [19,20]. Moreover, updated data of Antonarakis et al.’s study showed no correlation between the detection of ARV7 messenger in CTCs and primary resistance to taxane chemotherapy. In particular, if in ARV7-negative men taxanes and NHA could have comparable efficacy, in ARV7-positive men, chemotherapy with taxanes is associated with the best PSA response and PFS compared with enzalutamide or abiraterone [21]. 

More recently, another prospective multicenter study of circulating biomarkers (Prophecy) has validated ARV7 detection in CTCs as predictive of short PFS and OS in 118 men with mCRPC treated with new hormonal agents [22]. 

As a consequence, the identification of predictive biomarkers in the peripheral blood of patients, such as ARV7 status, could guide the choice of treatment in mCRPC, but the procedure of CTC isolation is only partially standardized. Nowadays, different methods are used for CTC isolation and ARV7 detection, so the availability of a standardized tool that can provide clinically relevant information and is easy enough to be reproduced outside of strictly research-focused laboratories represents a fundamental prerequisite. Thus, more findings are necessary to validate ARV7 detection as a predictive biomarker of resistance to NHA that can facilitate treatment selection.

In our prospective study, we used a commercially available kit to detect CTCs in blood samples collected from a real-world population of patients with mCRPC treated at our Institution with abiraterone or enzalutamide, as per clinical practice. The aim of our study was to validate a practical tool that is easy to reproduce in the laboratory and to determinate the impact of CTCs on clinical outcomes. An additional contribution of our study is that our data were generated during our routine clinical practice to offer a better picture of what happens in real life. We therefore feel that we are providing here a source of information useful to clinicians in guiding their treatment choice.

## 2. Results

### 2.1. Patient Characteristics

Between July 2015 and September 2017, a total of 37 patients treated with abiraterone or enzalutamide (26 and 11, respectively), as per clinical practice for mCRPC, were prospectively enrolled at our Institution to assess CTC status and the expression of AR and ARV7 (Table 1). Patients’ median age was 75 years (range of 68–80). All patients were Caucasian, had a confirmed adenocarcinoma histology, and were treated with standard androgen deprivation therapy (ADT). The Gleason score was ≥8 in 38% of patients (*n* = 14). Among all patients, 46% (*n* = 17) received local treatment on the primary tumor, of whom 76% (*n* = 13) underwent surgery and 23% (*n* = 4) received local radiotherapy. Concerning metastatic localization, 21 patients presented at baseline with only bone metastases and five patients with only visceral disease. Eleven patients presented with both visceral and bone involvement. The median follow-up (FU) was 25 months. Baseline characteristics are summarized in Table 1.

### 2.2. Baseline Liquid Biopsy Variables according to Patient Characteristics

The AdnaTest is a positive selection assay that employs beads functionalized with antibodies recognizing cell surface markers for CTC enrichments and multiplex RT-PCR of specific transcripts (*PSMA*, *PSA*, and *EGFR*) for CTC identification. As stated in the Methods section, samples were defined as CTC status positive (CTC^+ve^) when at least one of the investigated transcripts was above the expression threshold defined by the manufacturer. In 57% of our patients, CTC positivity was due to the concomitant high expression of *PSMA* and *PSA*, whereas in the remaining 43%, it was due to high expression of the *PSA* gene. *EGFR* was not detected in any of the samples [23]. Table 1 reports the expression of the biological variables under study (CTC status, AR, and ARV7) according to patients’ disease characteristics and demographics.

At baseline, 21/37 (57%) patients were defined as CTC^+ve^, 17/37 (46%) were AR status positive (AR^+ve^), and 9/37 (24%) were ARV7 status positive (ARV7^+ve^). Except for one case (which expressed the full-length receptor despite being classified as CTC status negative (CTC^−ve^)), AR and ARV7 were detected only in the blood of patients classified as CTC^+ve^. 

Age and years since diagnosis of mCRCPC were not significantly associated with CTC status or with AR and ARV7 detection.

Gleason scores, basal PSA levels, and previous local treatment on the primary tumor were associated with CTC detection and AR expression, but not with the expression of the variant ARV7. Conversely, the site of metastatic disease did not affect the expression of all our liquid biopsy variables. 

Patients with higher Gleason scores, higher basal PSA levels, and those who did not undergo any local treatments were more frequently CTC^+ve^ (*p* = 0.04, *p* = 0.009, and *p* = 0.005, respectively). Also, in the case of AR, the positive association with basal PSA reached statistical significance as observed for CTCs, but the association with Gleason score did not reach statistical significance (*p* = 0.40). Conversely, the detection of the splicing variant ARV7, both when evaluated in the overall population or in CTC^+ve^ samples only, was not found to be associated with the clinical variables correlated with disease aggressiveness and burden. 

Metastatic localization did not affect the CTC status, and neither the detection of AR and ARV7 nor differences in the distribution of biological variables were observed between patients treated with abiraterone versus enzalutamide (Table 1). Figure S1 reports the workflow of the analyses performed to test the clinical relevance of liquid biopsy variables.

### 2.3. Best Treatment Response according to Liquid Biopsy Variables

Treatment response, as a function of biological variables, is reported in Table 2.

Radiological response was evaluable for 35 patients. Among the evaluable cases, 43% (15/35) of patients were classified as responders by the evidence of complete response, stable disease, or partial response at CT or bone scan.

The best radiological response rate dropped from 66% to only 25% (5/20) in patients defined as CTC^−ve^ and CTC^+ve^, respectively (*p* = 0.02). Similarly, the detection of AR and ARV7 was associated with progression under treatment in 82% and 87% of patients, respectively. Whereas among ARV7 status negative (ARV7^−ve^) patients, progression and response (as best response) were observed in equal numbers of patients, when considering the CTCs and ARV7 together, 75% of CTC^+ve^/ARV7^−ve^ patients presented a progression as best response.

When considering the PSA response, associations between liquid biopsy variables and treatment best responses were in line with radiological responses but did not reach statistical significance. 

### 2.4. Time-Dependent Analyses of Liquid Biopsy Variables

Time-dependent analyses were run considering the impact of each liquid biopsy variable on PSA-PFS, rPFS, and OS. At a median follow-up of 25 months, 71% (25/35) of patients experienced a radiologically documented progression. Progression was strongly associated with CTC positivity (*p* = 0.0001). Thus, CTC positivity held a positive prediction value since all CTC^+ve^ patients experienced progression, but it had a weaker negative predictive value since only 62% of patients scored as CTC^−ve^ were progression-free.

**Figure 1:**
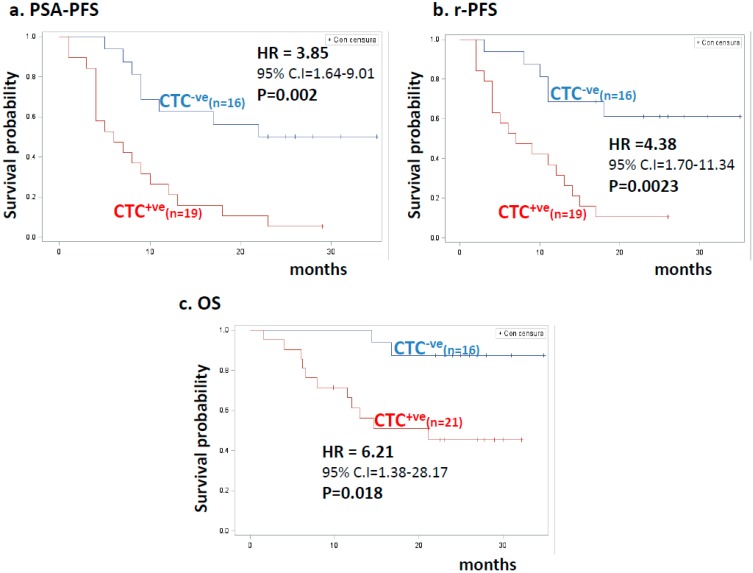
Kaplan–Meier estimates for clinical outcome in patients stratified by CTC status. (**a**) PSA-progression-free survival (PSA-PFS) according to CTC status. Blue and red lines indicate 16 CTC-negative (CTC^−ve^) and 19 CTC-positive (CTC^+ve^) patients, respectively. HR values, 95% confidence interval (CI), number of patients, and statistical significance are reported in the inset. (**b**) Radiologic progression-free survival (rPFS) according to CTC status. Blue and red lines indicate 16 CTC-negative (CTC^−ve^) and 19 CTC-positive (CTC^+ve^) patients, respectively. HR values, 95% confidence interval (CI), number of patients, and statistical significance are reported in the inset. (**c**) Overall survival (OS) according to CTC status. Blue and red lines indicate 16 CTC-negative (CTC^−ve^) and 21 CTC-positive (CTC^+ve^) patients, respectively. HR values, 95% confidence interval (CI), number of patients, and statistical significance are reported in the inset.

Patients with CTC^+ve^ blood samples showed significantly reduced PSA-PFS, rPFS, and OS compared with men with CTC^−ve^ blood samples (median times to progression or death: 5.85 vs. 22.34 months, *p* = 0.002, for PSA-PFS; 7.43 vs. 29.59 months, *p* = 0.0023, for rPFS; and 14.60 vs. >24 months, *p* = 0.018, for OS). For CTC^+ve^ patients, the two-year hazard risk (HR) for relapse was 3.85-fold higher compared with patients with CTC^−ve^ blood samples at baseline. Similar data were observed for rPFS. The HR for survival was even stronger (HR = 6.21). Kaplan–Meier plots, HR values with 95% confidence interval (CI), and statistical significance are reported in Figure 1.

**Figure 2:**
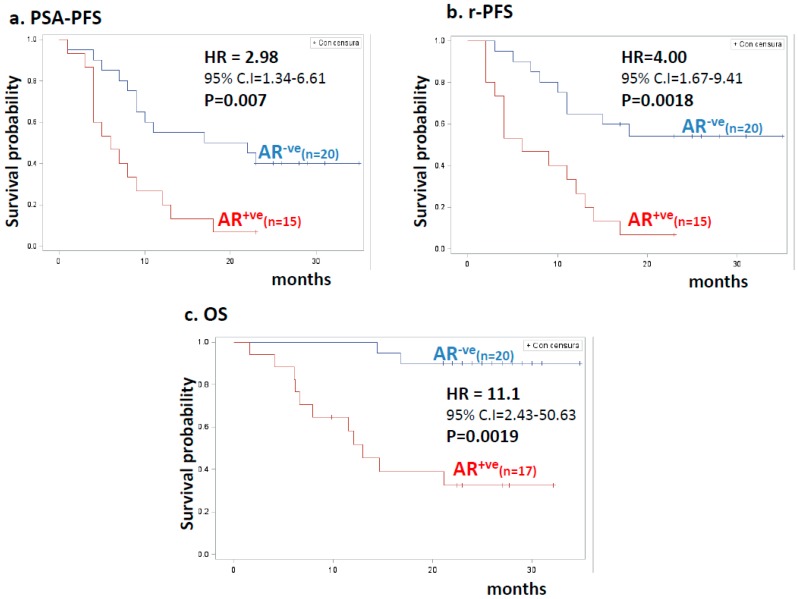
Kaplan–Meier estimates for clinical outcome in patients stratified by AR detection. (**a**) PSA-progression-free survival (PSA-PFS) according to AR detection. Blue and red lines indicate 20 AR-negative (AR^−ve^) and 15 AR-positive (AR^+ve^) patients, respectively. HR values, 95% confidence interval (CI), number of patients, and statistical significance are reported in the inset. (**b**) Radiologic progression-free survival (rPFS) according to AR status. Blue and red lines indicate 20 AR-negative (AR^−ve^) and 15 AR-positive (AR^+ve^) patients, respectively. HR values, 95% confidence interval (CI), number of patients, and statistical significance are reported in the inset. (**c**) Overall survival (OS) according to AR status. Blue and red lines indicate 20 AR-negative (AR^−ve^) and 17 CTC-positive (AR^+ve^) patients, respectively. HR values, 95% confidence interval (CI), number of patients, and statistical significance are reported in the inset.

The positive detection of AR was also associated with shorter rPFS, PSA-PFS, and OS. The two-year HR for recurrence in patients classified as AR^+ve^ was 3–4-fold higher compared with AR status negative (AR^−ve^) cases, with an 11-fold higher risk of death. Half of patients with AR^+ve^ blood samples relapsed within six months (both when evaluated radiologically or biochemically), whereas the median progression time was at least three times longer for those classified as AR^−ve^. Similarly, the median OS was double in AR^−ve^ patients compared with AR^+ve^ (Figure 2).

**Figure 3:**
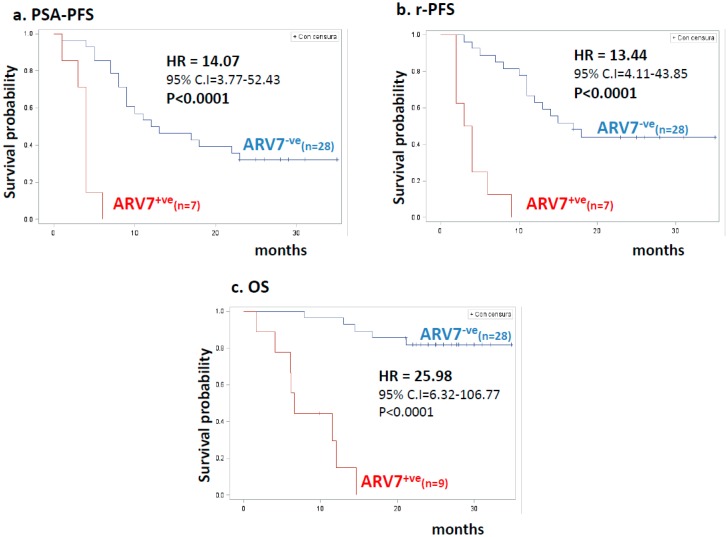
Kaplan–Meier estimates for clinical outcome in patients stratified by ARV7 detection. (**a**) PSA-progression-free survival (PSA-PFS) according to ARV7 detection. Blue and red lines indicate 28 ARV7-negative (ARV7^−ve^) and seven ARV7-positive (ARV7^+ve^) patients, respectively. HR values, 95% confidence interval (CI), number of patients, and statistical significance are reported in the inset. (**b**) Radiologic progression-free survival (rPFS) according to AR status. Blue and red lines indicate 28 ARV7-negative (ARV7^−ve^) and seven ARV7-positive (ARV7^+ve^) patients, respectively. HR values, 95% confidence interval (CI), number of patients, and statistical significance are reported in the inset. (**c**) Overall survival (OS) according to ARV7 status. Blue and red lines indicate 28 ARV7-negative (ARV7^−ve^) and nine ARV7-positive (AR^+ve^) patients, respectively. HR values, 95% confidence interval (CI), number of patients, and statistical significance are reported in the inset.

For ARV7 (Figure 3), the relative risks were much higher than those calculated for the full-length receptor, with similar HR values for biochemical or radiologic progression and definitely very high HRs for death (HR = 26, 95% CI 6.32–106.7, *p* < 0.0001).

Overall, positivity for CTCs, as well as for AR or ARV7, strongly predicted progression. The prognostic role of ARV7 in survival was particularly strong, as none of the AR7^+ve^ patients were alive at two years from the start of treatment.

**Figure 4:**
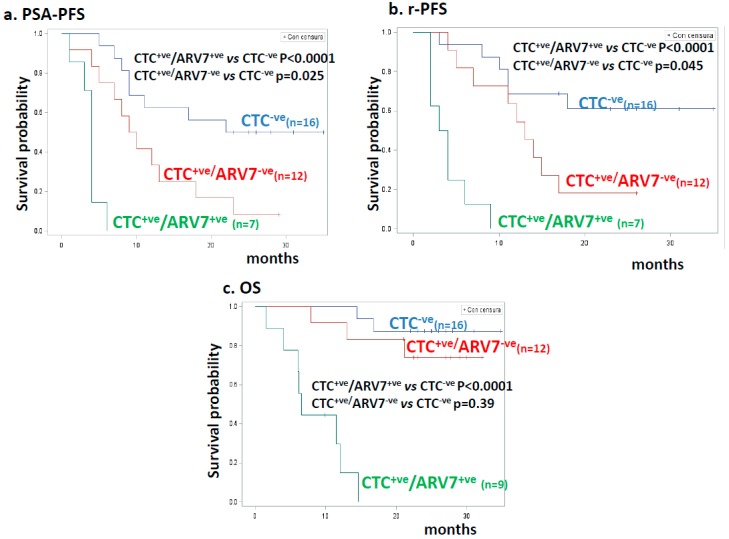
Kaplan–Meier estimates for clinical outcome in patients stratified by CTC and ARV7 detection. (**a**) PSA-progression-free survival (PSA-PFS) according to ARV7 detection. Blue, red, and green lines indicate 16 CTC-negative (CTC^−ve^), 12 CTC-positive/ARV7-negative (CTC^+ve^/ARV7^−ve^), and seven CTC-positive/ARV7-positive (CTC^+ve^/ARV7^+ve^) patients, respectively. HR values, 95% confidence interval (CI), number of patients, and statistical significance are reported in the inset. (**b**) Radiologic progression-free survival (rPFS) according to AR status. Blue, red, and green lines indicate 16 CTC-negative (CTC^−ve^), 12 CTC-positive/ARV7-negative (CTC^+ve^/ARV7^−ve^), and seven CTC-positive/ARV7-positive (CTC^+ve^/ARV7^+ve^) patients, respectively. HR values, 95% confidence interval (CI), number of patients, and statistical significance are reported in the inset. (**c**) Overall survival (OS) according to ARV7 status. Blue, red, and green lines indicate 16 CTC-negative (CTC^−ve^), 12 CTC-positive/ARV7-negative (CTC^+ve^/ARV7^−ve^), and nine CTC-positive/ARV7-positive (CTC^+ve^/ARV7^+ve^) patients, respectively. HR values, 95% confidence interval (CI), number of patients, and statistical significance are reported in the inset.

The best risk stratification was obtained by considering the association of the two variables CTC and ARV7, as illustrated in Figure 4. Indeed, the addition of the ARV7 variable to CTC positivity was able to identify the patients with the shortest survival; OS did not significantly differ between CTC^−ve^ patients and those with CTC^+ve^/ ARV7^−ve^ blood samples (*p* = 0.39). The risk of progression for CTC^+ve^ patients lacking ARV7 was intermediate, between the CTC^−ve^ ones (low risk) and the CTC^+ve^/ARV7^+ve^ ones (high risk).

### 2.5. Prognosis according to Basal PSA Levels and Liquid Biopsy Variables

As expected, when analyzed as a continuous variable, the increase in PSA basal levels significantly impacted both rPFS and OS (HR = 1.007, 95% CI 1.003–1.012, *p* = 0.002; HR = 1.009, 95% CI 1.003–1.014, *p* = 0.0037). 

**Figure 5:**
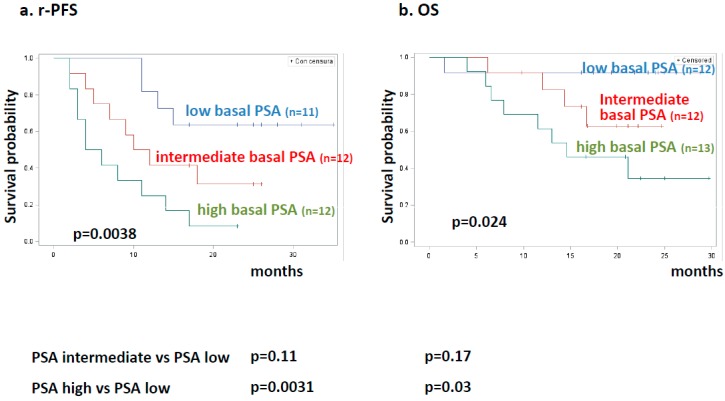
Kaplan–Meier estimates for clinical outcome in patients stratified by basal PSA levels. (**a**) Radiologic progression-free survival (rPFS) in patients stratified for basal PSA levels by tertiles. Blue, red, and green lines represent 11 patients with low, 12 intermediate, and 12 high basal PSA levels, respectively. (**b**) Overall survival (OS) in patients stratified for basal PSA levels by tertiles. Blue, red, and green lines represent 12 patients with low, 12 intermediate, and 13 high basal PSA levels, respectively.

Figure 5 reports Kaplan–Meier estimates for rPFS and OS in patients stratified for PSA basal levels by tertiles (PSA ≤ 18 ng/mL vs. PSA 18–63 vs. PSA > 63). Also, as a categorical variable, PSA significantly impacted both rPFS and OS. However, patients with “intermediate” PSA levels did not significantly differ from those with low PSA levels, both for rPFS and for OS (*p* = 0.11 and *p* = 0.15, respectively). rPFS and OS were instead significantly different between patients with high PSA levels versus those with low levels (*p* = 0.0031, *p* = 0.03, respectively).

This result prompted us to analyze the role of liquid biopsy variables within patient subgroups stratified by basal PSA levels. Results are reported in Table S2. At two years, within the subgroup of men with low PSA levels, CTC, AR, and ARV7 did not significantly contribute to predict rPFS. Similar data were obtained for the patient subgroup with high PSA levels, except for the detection of ARV7. Indeed, at two years, none of the patients with high ARV7 levels were progression-free (*p* = 0.01). 

Conversely, in patients with intermediate PSA levels, radiologic progression was significantly impacted by CTC, AR, and ARV7 detection. Data for rPFS by PSA levels are reported in Table S2.

Table S3 reports results for OS. Only ARV7 detection had a statistically significant impact among patients with intermediate PSA levels, whereas in the patient subgroup with high PSA levels, positive detection of all the studied variables significantly reduced survival rates.

### 2.6. Overall Survival as a Function of Other Treatments after ADT 

Patients’ overall survival is not only impacted by antiandrogen treatment but also by subsequent treatment lines. We therefore separately analyzed the role of our biological variables in OS in patients receiving or not taxane-based treatment after enzalutamide or abiraterone. Results are summarized in Table 3.

In the subgroup of 24 patients not receiving taxanes, HR values for CTC status positivity, AR and ARV7 detection, and their combination are not only statistically significant but are higher than those reported for the overall population (Figure 1, Figure 2, Figure 3 and Figure 4). Conversely, the administration of taxanes after antiandrogen therapy seems to abrogate the prognostic relevance of CTC and AR in OS. Only the expression of ARV7 maintains a statistically significant negative impact on OS. 

## 3. Discussion

Enzalutamide and abiraterone have been shown to improve PFS and OS in mCRPC patients [1,2,3,4]. However, the inhibition of AR activity is ineffective in around 20–40% of patients due to primary or secondary resistance [5]. In most cases, a constitutive activation of receptor splice variants, including the variant ARV7, is involved in determining this resistance. The ARV7 variant encodes a truncated androgen receptor protein that lacks the ligand-binding domain (target of enzalutamide and abiraterone), generating a ligand-independent activation [6,7,8,9]. 

As a consequence, identifying biomarkers able to predict resistance to treatment with NHA could be crucial to guide treatment choice for mCRPC patients. CTCs have been extensively used as prognostic biomarkers [13,14,24], and although their prognostic value may be of paramount clinical interest, CTC detection in mCRPC is not currently included in daily clinical practice since it has not been validated as a decision-making test. Nonetheless, the molecular profile of CTCs can be exploited as a reflection of the molecular profile of the disease, thus providing a way to evaluate treatment-prediction biomarkers. 

Several studies have shown that the detection of the ARV7 variant in patients’ CTCs was associated with treatment failure in men with high-risk mCRPC treated with NHA [19,20,22,25,26,27]. Currently, different technical approaches are used to detect CTCs and ARV7, and there is still a need for simple, widely available standardized tools for extending personalized biomarker-driven treatments to all patients and facilitating treatment selection in daily clinical practice. The availability of a standardized tool is, in fact, a fundamental prerequisite to provide clinically relevant information. Most studies, however, use proprietary tests, thus hindering comparisons. Indeed, for ease of comparison among results obtained from different centers, in the multicentric Prophecy trial, AR and ARV7 transcripts were centrally measured in a reference laboratory using a Johns Hopkins mRNA assay [22].

In our prospective observational study, we identified a real-world population of 37 patients with mCRPC treated at INT with abiraterone or enzalutamide, as per clinical practice. We collected patients’ samples before starting treatment to evaluate the impact on clinical outcomes of CTC, AR, and ARV7 expression. For CTC enrichment, we used the AdnaTest ProstateCancerSelect kit, originally employed by Antonarakis et al. and adopted in the multicentric Prophecy trial [22]. For detecting AR and ARV7 expression, we employed a commercially available kit, which uses aPCR assay recently standardized and validated in Italy [28]. The chosen assay proved to be easily reproducible and offered a robust tool with high sensitivity and specificity for the detection and quantification of ARV7 and AR. In fact, consistent with previous studies conducted with the AdnaTest, we detected CTC positivity in 57% of cases (21/37 patients), AR positivity in 46% (17/37 patients), and ARV7 positivity in 24% (9/37 patients) [1,2,3,4,5,6,7,8,9,19,20,21,25,26,27,29,30,31].

In the present study, Gleason score, basal PSA levels, and previous local treatment on primary tumor showed a positive relationship with CTC detection and AR expression, while the expression of ARV7 was independent of clinical variables. In contrast, data available in the literature reported that patients with higher Gleason scores and higher PSA levels were more likely to present positivity not only for CTC but also for AR and ARV7 expression, suggesting that CTC and AR/ARV7 status identifies patients with more advanced disease and therefore poorer prognosis. Moreover, several retrospective studies showed that androgen receptor variants are expressed more often in men with metastatic CRPC than in metastatic hormone-sensitive disease, assuming a causal role of these variants in mediating progression to a castration-resistant phase. Thus, ARV7 could be a marker of advanced disease or higher tumor burden and poor prognosis [19,20]. Furthermore, a recent study determined whether the detection of CTCs adjusted for imbalances in baseline characteristics impacts survival, demonstrating no difference in OS between CTC+/ARV7+ and CTC+/ARV7-patients [29]. One potential limitation of CTC ARV7 testing is its dependence on the presence and count of CTCs, which could confound outcome analyses [19].

To our knowledge, our study is the first to report a significantly higher probability of detecting CTCs and AR positivity in patients who did not receive treatment for the primary tumor (i.e., radical prostatectomy or radical radiotherapy).

In the current study, Kaplan–Meier estimates showed that positivity for CTC, AR, and ARV7 expression at baseline was significantly associated with poorer rPFS, PSA-PFS, and OS, as reported by Antonarakis et al. and confirmed by successive studies [19,20,21,22,25,26,27,29]. The expression of ARV7, in particular, identified patients at a higher risk of death, confirming its prognostic role. An insignificant difference in OS was observed in patients with negative CTC status compared to patients with positive CTC status but negative ARV7, supporting the hypothesis that ARV7 could actually play an important role in resistance to NHA. 

CTC positivity, AR, and ARV7 expression showed a strong negative association with radiological and PSA progression (defined by RECIST 1.1. and PCWG criteria, respectively). At baseline, the detection of AR and ARV7 was associated with progression to NHA in 82% and 87% of patients, respectively. Consistent with the literature, the presence of ARV7 in CTCs could predict a poor response to AR inhibition, whereas its absence in CTCs does not uniformly predict outcome. Our results showed a statistically significant difference for the best PSA response but not for the best radiological response. In clinical practice, discordance between PSA and radiological responses is often observed. These data should, however, be interpreted with caution due to the low number of patients included in the analysis. Moreover, the fact that our data reflect a real-world population may also have contributed to the results. 

Updated data of Antonarakis et al.’s study showed no association between the detection of ARV7 messenger in CTCs and primary resistance to taxane chemotherapy [21]. In particular, ARV7-positive men receiving chemotherapy with taxanes showed better PSA response and better PFS compared with NHA [21]. Unlike Antonarakis et al., we are not performing here a direct comparison between NHA and taxanes and cannot draw any conclusions on the role of ARV7 in predicting resistance to chemotherapy.

CTCs and ARV7 status maintained a prognostic role throughout subsequent treatment lines in the entire cohort of patients and after stratification based on tertiles of basal PSA levels. Furthermore, patients with a positivity of both CTCs and ARV7 had a worse outcome than CTC-positive and ARV7-negative patients or CTC-negative patients. These results are in line with the Prophecy trial, which validated ARV7 detection as a predictive biomarker of short PFS and OS [21].

Thus, our study, in line with the literature, confirmed the association between higher CTCs and poorer prognosis, providing useful information for daily clinical practice. The main limitation of our study was the limited sample size. However, we reported a reflection of real-life clinical practice, which adds more value to our work.

Having acknowledged the predictive relevance of ARV7 and its direct involvement in causing resistance to NHA, new drugs which specifically target the ARV7 receptor have been identified and tested. One of them, niclosamide, has recently been introduced into clinical trials after positive results in preclinical studies [32]. 

ARV7 monomers have been described as not transcriptionally functional proteins [33]; therefore, interfering in their heterodimerization process could be a possible mechanism to develop therapeutic approaches [34].

However, recent insights into the mechanism by which ARV7 promotes CRPC offer additional treatment opportunities. In particular, a study by Cato et al. suggests that ARV7 is transcriptionally active, and unlike the full-length receptor, the splicing variant preferentially exerts repressive effects on gene transcription. Indeed, although AR and ARV7 can potentially heterodimerize and colocalize on the DNA as previously suggested [33], ARV7 itself preferentially binds corepressors rather than coactivators. Among the repressed genes, the same authors identified four specific genes that inhibit cell growth (thus explaining the negative prognostic relevance of ARV7) and that, when present, may be possible biomarkers for optimal ARV7 inhibition, thus offering a new tool for monitoring treatment efficacy.

Overall, five years since the first reports on the role of ARV7 as a prognostic and treatment response predictive biomarker, the role of ARV7 has been constantly confirmed by new reports and also extended to other malignances such as salivary duct carcinoma (SDC), where AR is an important driver [35]. Recent evidence, however, has shown that the detection of ARV7 alone could be insufficiently accurate to predict response to NHA and simultaneous detection of other variants might improve prediction [30].

## 4. Materials and Methods 

### 4.1. Patients

Between July 2015 and September 2017, we identified 37 patients treated with NHA for mCRPC, defined as a metastatic progressive disease despite androgen-deprivation therapy with “castration levels” of serum testosterone (<50 ng per deciliter; 1.73 nmol per liter). Prior treatment with chemotherapy for hormone-sensitive disease was permitted, as well as prior treatment with abiraterone or enzalutamide if it was planned to administer the alternative agent (i.e., prior abiraterone use in enzalutamide-treated patients and vice versa). Previous therapies are shown in Table S1 in the Supplementary Materials. All patients gave their informed consent at the start of treatment, allowing the use of residual material for research purposes. Since the study included patients recruited during daily clinical practice, no approval by the Local Ethical Board was required.

### 4.2. CTC Assessment

Peripheral whole blood samples were collected in K_3_EDTA BD Vacutainer tubes at baseline corresponding with the time of the start of treatment with enzalutamide or abiraterone. CTC detection was performed on blood samples obtained after withdrawal for other tests to minimize the risk of contamination with epithelial skin cells during puncture. Enrichment for CTCs was performed using the AdnaTest ProstateCancerSelect kit (AdnaGen, AG, Langenhagen, Germany) by incubating 5 mL of whole blood with 100 μL of magnetic beads coated with antibodies against the epithelial and tumor-associated antigens EpCAM and Erb-B2. The AdnaMag-L magnetic particle concentrator was used for bead recovery, and cell lysates prepared according to the manufacturer’s instructions were stored at −20 °C for up to 14 days before mRNA isolation and molecular analysis using the AdnaTest ProstateCancerDetect kit. 

The expression of *PSMA*, *PSA*, and *EGFR* (i.e., epithelial and tumor-specific markers) and *ACTB*, as a control, was assessed by semiquantitative multiplex PCR using the PrimerMix provided in the AdnaTest ProstateCancerDetect kit. The presence and concentration of the above PCR products were evaluated by capillary gel electrophoresis by the Agilent 2100 Bioanalyzer (Agilent Technologies, Santa Clara, CA, USA) using the DNA 1000 kit (Agilent Technologies). Samples were classified as CTC status positive (CTC^+ve^) when at least one of the tumor-specific markers (*PSMA*, *PSA*, and *EGFR*) was above the threshold defined by the manufacturer (0.10 ng/µL). Only samples with *ACTB* concentrations of ≥3 ng/µL were considered as evaluable for CTC. Expression of *AR* and *ARV7* was evaluated on the same cDNA samples prepared for CTC status determination described above using the AR-V7 RTPCR kit (AR-V7 assay RT-PCR, Bird, Monteriggioni, Italy) according to the manufacturer’s instructions. The kit allows an absolute quantification, and the threshold for AR and ARV7 positivity was set at ≥10 copies/mL. CTC, AR, and ARV7 determinations were run, and results were analyzed without knowledge of clinical data.

### 4.3. Clinical Outcomes

This study was designed to determine the impact of each liquid biopsy variable on three distinct clinical outcomes: OS, defined as the time from the date of the start of treatment to death from any cause; PSA-PFS, defined as freedom time from PSA progression; and rPFS, defined as the time from the start of treatment to the first objective evidence of radiographic disease progression. PSA progression was defined by three or more consecutive increases of 25% or more above the nadir (and by ≥2 ng per milliliter) one week apart, while PSA response was defined by ≥50% decline in PSA level from baseline, maintained for ≥4 weeks at any time after the initiation of therapy. Radiographic progression was defined by an increase of ≥20% in the sum of the diameters of soft-tissue target lesions on a CT scan according to the Response Evaluation Criteria in Solid Tumors criteria (RECIST 1.1) or by the evidence of ≥2 new bone lesions on a bone scan according to PCWG3 criteria or death [36,37]. We also evaluated the association between the best radiological response (defined by the evidence of complete response, stable disease, or partial response at CT or bone scan), the best PSA response (maximal percentage decrease in PSA level from baseline), and liquid biopsy variables. 

### 4.4. Statistical Analysis

Kaplan–Meier curves were plotted using the cut-offs reported above each biological variable and their combinations. Survival differences were estimated by using the log-rank test. Univariable Cox proportional hazards regressions, as implemented in the SAS package (version 9.4, SAS Institute Inc., Cary, NC, USA), were used evaluate two-year HRs.

## 5. Conclusions

In conclusion, our study of a real-world population of patients with mCRPC, although limited by the small sample size, confirmed the association between higher CTCs and poorer prognosis and the importance of the truncated ligand-independent variant ARV7 in promoting androgen-independent cell proliferation in CRPC patients, as has been extensively reported in the literature. The commercially available kit used to perform the analysis was confirmed to be reliable and reproduced results consistent with already-published data.

Thus, CTC, AR, and ARV7 detection could be useful in clinical practice to predict worse outcomes and to guide future treatment choices with new targeted agents [6,7,8,9].

## Figures and Tables

**Table 1:** Liquid biopsy variables according to patient baseline demographic and clinical characteristics.

Baseline Characteristics	All	CTC^+ve^	CTC^−ve^	*p*	AR^+ve^	AR^−ve^	*p*	ARv7^+ve^	ARv7^−ve^	*p*	CTC^+ve^/ARv7^+ve^	CTC^+ve^/ARv7^−ve^	*p*
**Total**	37	21	16	/	17	20	/	9	28		9	12	
**Age**	75.3	75.3	74.6	0.37	75.1	76.6	0.84	78.5	75.1	0.64	78.5	75.1	0.97
**Months since diagnosis of metastatic castration-resistant prostate cancer (mCRPC)**	0.73	1.27	0.57	0.365	1.58	0.47	0.13	2.5	0.72	0.97	2.5	1.0	0.80
**Gleason sum at diagnosis**				0.04			0.40			0.25			0.08
≤7	9	1	8		2	7		0	9		0	1	
≥8	14	8	6		6	8		3	11		3	5	
NA	14												
**Metastases at diagnosis**				0.73			0.47			0.50			0.31
Bone	21	13	8		11	10		6	15		6	7	
Viscera	5	3	2		1	4		0	5		0	3	
Bone+viscera	11	5	6		6	5		3	8		3	2	
**Local treatment**				0.005			0.009			0.295			1.00
Surgery	13	4	9		4	9		2	11		2	2	
RT^1^	4	1	3		0	4		0	4		0	1	
None	20	16	4		14	6		7	13		7	9	
**Basal PSA**				0.09			0.02			0.21			0.85
≤18	12	4	8		3	9		1	11		1	3	
18–63	12	7	5		4	8		3	9		3	4	
≥63	13	10	3		10	3		5	8		5	5	
**Systemic treatment**				0.28			0.29			1.0			1.0
Abiraterone	26	13	13		11	15		6	20		6	7	
Enzalutamide	11	8	3		7	4		3	8		3	5	

^1^ Radiotherapy.

**Table 2:** Liquid biopsy variables according to best treatment response.

Treatment Outcome	All	CTC^+ve^	CTC^−ve^	*p*	AR^+ve^	AR^−ve^	*p*	ARv7^+ve^	ARv7^−ve^	*p*	CTC^+ve^/ARv7^+ve^	CTC^+ve^/ARv7^−ve^	*p*
**Best radiologic response**				0.019			0.006			0.10			0.60
CR^1^+PR^2^+SD^3^	15	5	10		3	12		1	14		1	4	
PD^4^	20	15	5		14	6		7	13		7	8	
NA	2												
**Best PSA response**				0.07			0.13			0.12			0.07
CR+PR+SD	25	11	14		9	16		2	23		2	9	
PD	10	8	2		7	3		5	5		5	3	
NA	2												

^1^ Complete Response, ^2^ Partial Response, ^3^ Stable Disease, ^4^ Progressive Disease.

**Table 3:** Univariable analysis for OS as a function of biological variables in patients treated or not with taxanes.

Biological Variable		No Taxanes Post A/E^1^			Taxanes Post A/E		
		Overall Survival (*n* = 24; 10 Events)			Overall Survival (*n* = 13; 3 Events)		
		HR^2^	95% CI	*p* value	HR	95% CI	*p* value
**CTC**	CTC^+ve^ *vs* CTC^−ve^	18.61	2.30–150.27	0.006	0.91	0.08–9.69	0.9
**AR**	ARV^+ve^ *vs* ARV^−ve^	18.61	2.31–150.27	0.006	3.16	0.32–39.31	0.3
**ARV7**	ARV7^+ve^ *vs* ARV7^−ve^	33.96	3.84–299.88	0.0015	17.20	1.52–194.13	0.021

^1^ Abiraterone/Enzalutamide, ^2^ Hazard Ratio.

## Supplementary Materials

The following are available online at https://www.mdpi.com/2072-6694/11/7/980/s1, Table S1: Treatment ongoing at time of CTC determination, Table S2: Two-year radiological progression-free survival rates according to basal PSA levels, Table S3: Two-year radiological progression-free survival rates according to basal PSA levels. Figure S1: Workflow of analyses.
