# Supplementary material for: Could Circulating Tumor Cells and ARV7 Detection Improve Clinical Decisions in Metastatic Castration-Resistant Prostate Cancer? The Istituto Nazionale dei Tumori (INT) Experience
Source: Cancers (Basel). 2019 Jul 13;11(7):980. doi: 10.3390/cancers11070980 (PMC6678845; doi:10.3390/cancers11070980)
Supplement: Supplementary file 1 [file cancers-11-00980-s001.zip › cancers-531463-SI/supplementaryMaterials/FigS1.pptx]

## Slide 1
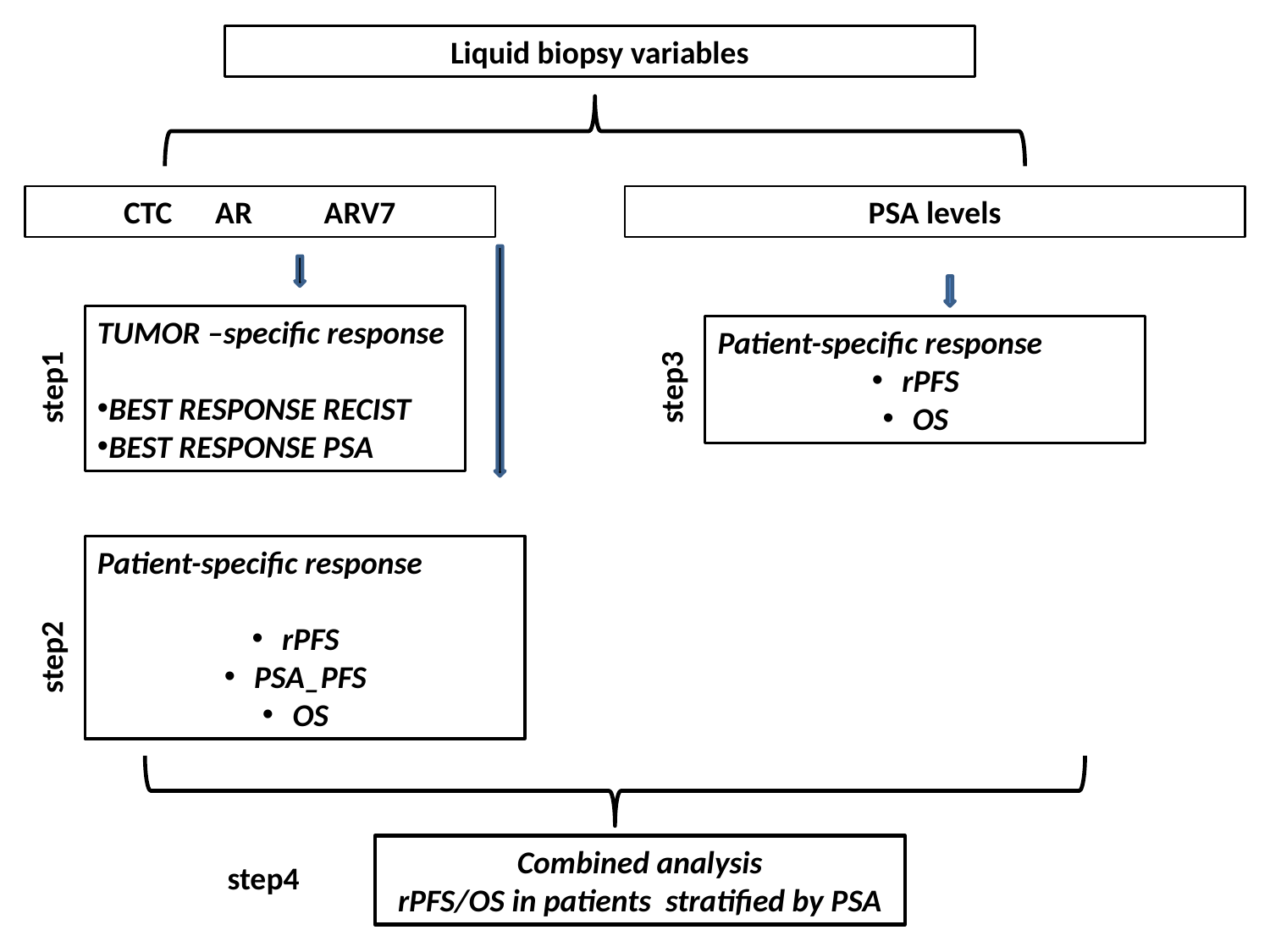

Liquid biopsy variables
CTC AR ARV7
PSA levels
TUMOR –specific response
BEST RESPONSE RECIST
BEST RESPONSE PSA
Patient-specific response
rPFS
OS
step1
step3
Patient-specific response
rPFS
PSA_PFS
OS
step2
Combined analysis
rPFS/OS in patients stratified by PSA
step4
