# Supplementary material for: Could Circulating Tumor Cells and ARV7 Detection Improve Clinical Decisions in Metastatic Castration-Resistant Prostate Cancer? The Istituto Nazionale dei Tumori (INT) Experience
Source: Cancers (Basel). 2019 Jul 13;11(7):980. doi: 10.3390/cancers11070980 (PMC6678845; doi:10.3390/cancers11070980)
Supplement: Supplementary file 1 [file cancers-11-00980-s001.zip › cancers-531463-SI/supplementaryMaterials/Table S1.docx]

Table S1: Treatment ongoing at time of CTC determination

| **Treatment** | **All patients (n=37)** |
| --- | --- |
| Abiraterone | 26 (70)  11 (30) |
| Enzalutamide |  |
| **Treatment Line** |  |
| - 1st | 31 (84) |
| - 2nd | 5 (13) |
| - other | 1 (3) |
| **Abiraterone line** |  |
| - 1st | 24 (92) |
| - 2nd | 2 (8) |
| - 3rd | 0 (0) |
| **Enzalutamide line** |  |
| - 1st | 7 (64) |
| - 2nd | 3 (27) |
| - 3rd | 1 (9) |
| **Prior use of abiraterone for mCRPC** |  |
| - yes | 3 (27) |
| - no | 8 (73) |
| **Prior use of enzalutamide for mCRPC** |  |
| - yes | 2 (8) |
| - no | 24 (92) |
