# Supplementary material for: Could Circulating Tumor Cells and ARV7 Detection Improve Clinical Decisions in Metastatic Castration-Resistant Prostate Cancer? The Istituto Nazionale dei Tumori (INT) Experience
Source: Cancers (Basel). 2019 Jul 13;11(7):980. doi: 10.3390/cancers11070980 (PMC6678845; doi:10.3390/cancers11070980)
Supplement: Supplementary file 1 [file cancers-11-00980-s001.zip › cancers-531463-SI/supplementaryMaterials/Table S2.docx]

**Table S2. Two-year radiological progression-free survival rates according to basal PSA levels**

|  | | **Low pre-treatment PSA** | | | **Intermediate pre-treatment PSA** | | | **High pre-treatment PSA** | | |
| --- | --- | --- | --- | --- | --- | --- | --- | --- | --- | --- |
|  | | **Progression-free survival**  **(n=11; 4 events)** | | | **Progression-free survival**  **(n=12; 7 events)** | | | **Progression-free survival**  **(n=12; 11 events)** | | |
|  |  | **#** | **%** | **p value** | **#** | **%** | **p value** | **#** | **%** | **p value** |
| **CTC** | negative | 8 | 75 | 0.32 | 5 | 53 | 0.025 | 3 | 33 | 0.33 |
|  | positive | 3 | 33 |  | 7 | 14 |  | 9 | 0 |  |
|  |  |  |  |  |  |  |  |  |  |  |
| **AR** | negative | 9 | 67 | 0.70 | 8 | 47 | 0.028 | 3 | 33 | 0.33 |
|  | positive | 2 | 50 |  | 4 | 0 |  | 9 | 0 |  |
|  |  |  |  |  |  |  |  |  |  |  |
| **AR v7** | negative | 11 | 64 |  | 9 | 42 | 0.005 | 7 | 14 | 0.01 |
|  | positive |  |  |  | 3 | 0 |  | 5 | 0 |  |
|  | negative |  |  |  |  |  |  |  |  |  |
| **CTC&ARv7** | CTC^-ve^ | 8 | 75 | 0.32 | 5 | 53 | 0.011 | 3 | 33 | 0.036 |
|  | CTC^+ve^/ARv70^-ve^ | 3 | 33 |  | 4 | 25 |  | 4 | 0 |  |
|  | CTC^+ve^/ARv70^+ve^ |  |  |  | 3 | 0 |  | 5 | 0 |  |
