# Supplementary material for: Could Circulating Tumor Cells and ARV7 Detection Improve Clinical Decisions in Metastatic Castration-Resistant Prostate Cancer? The Istituto Nazionale dei Tumori (INT) Experience
Source: Cancers (Basel). 2019 Jul 13;11(7):980. doi: 10.3390/cancers11070980 (PMC6678845; doi:10.3390/cancers11070980)
Supplement: Supplementary file 1 [file cancers-11-00980-s001.zip › cancers-531463-SI/supplementaryMaterials/Table S3.docx]

**Table S3. Two-year overall survival rates according to basal PSA levels**

|  | | | **Low pre-treatment PSA** | | | **Intermediate pre-treatment PSA** | | | **High pre-treatment PSA** | | |
| --- | --- | --- | --- | --- | --- | --- | --- | --- | --- | --- | --- |
|  | |  | **Overall survival**  **(n=12; 1 event)** | | | **Overall survival**  **(n=12; 4 events)** | | | **Overall survival**  **(n=13; 8 events)** | | |
|  | | | **#** | **%** | **p value** | **#** | **%** | **p value** | **#** | **%** | **p value** |
| **CTC** | negative | | 8 | 100 | 0.16 | 5 | 60 | 0.9 | 3 | 100 | 0.04 |
|  | positive | | 4 | 75 |  | 7 | 69 |  | 10 | 20 |  |
|  |  | |  |  |  |  |  |  |  |  |  |
| **AR** | negative | | 9 | 100 | 0.08 | 8 | 75 | 0.11 | 3 | 100 | 0.04 |
|  | positive | | 3 | 67 |  | 4 | 37 |  | 10 | 20 |  |
|  |  | |  |  |  |  |  |  |  |  |  |
| **AR v7** | negative | | 11 | 100 |  | 9 | 78 | 0.002 | 8 | 62 | 0.0027 |
|  | positive | | 1 | 0 |  | 3 | 0 |  | 5 | 0 |  |
|  |  | |  |  |  |  |  |  |  |  |  |
| **CTC&ARv7** | CTC negative | | 8 | 100 | 0.004 | 5 | 60 | 0.004 | 3 | 100 | 0.006 |
|  | CTC+/ARv70- | | 3 | 100 |  | 4 | 100 |  | 5 | 40 |  |
|  | CTC+/ARv70+ | | 1 | 0 |  | 3 | 0 |  | 5 | 0 |  |
